# Neuropsychological correlates of ADHD: indicators of different attentional profiles among youth with sluggish cognitive tempo

**DOI:** 10.3389/frcha.2023.1208660

**Published:** 2023-07-18

**Authors:** Beth Krone, Anne-Claude V. Bédard, Kurt Schulz, Iliyan Ivanov, Mark A. Stein, Jeffrey H. Newcorn

**Affiliations:** ^1^ Department of Psychiatry, Icahn School of Medicine at Mount Sinai, New York, NY, United States; ^2^ Department of Applied Psychology and Human Development, Ontario Institute for Studies in Education, University of Toronto, Toronto, ON, Canada; ^3^ Department of Psychiatry and Behavioral Sciences and Pediatrics, University of Washington, Seattle, WA, United States

**Keywords:** attention network test (ANT), Attention Deficit Hyperactivity Disorder (ADHD), sluggish cognitive tempo (SCT), cognitive disengagement syndrome, continuous performance test (CPT), phenotyping, objective measures, child psychiatric and psychological assessment

## Abstract

**Objective:**

This study examined the distinctiveness of Attention Deficit Hyperactivity Disorder—Inattentive (ADHD-I) and ADHD in context of Sluggish Cognitive Tempo (ADHD + SCT) utilizing the Attention Network Test (ANT) and Continuous Performance Test (CPT) as external validators. Due to the SCT characteristics of being sluggish, spacey, and slow to arouse, we hypothesized that SCT behavioral descriptors would be uniquely related to alerting/arousal mechanisms that the ANT is uniquely designed to capture, and that ADHD symptoms would be more highly associated with cognitive control on the CPT.

**Method:**

We examined associations between baseline ANT and CPT scores for *N* = 137 well-characterized, culturally and racially diverse youth with ADHD (*n* = 107) either medication naïve or washed out prior to testing and typically developing controls (*n* = 30) ages 6–17 years.

**Results:**

Presence and severity of SCT were associated with ANT Alerting (*r*^2^ = −.291, *p* = .005), but not with ANT Orienting, ANT Executive Control, or any CPT measures. There was a distinct association between the presence and severity of ADHD inattention symptoms with CPT *T*-scores for Commission Errors (*r*^2^ = .282, *p* = .002), Omission Errors (*r*^2^ = .254, *p* = .005), Variability (*r*^2^ = .328, *p* < .001), and Hit Rate SE (*r*^2^ = .272, *p* = .002), but not with other CPT or any ANT domain measures. All associations remained significant after Bonferroni correction.

**Conclusions:**

The small but enduring double dissociation, with ADHD-I symptom severity related to measures of cognitive and behavioral control measures on the CPT, and SCT symptom severity related to attentional processes underlying tonic arousal in preparation for cue detection on the ANT—provides the first objective evidence suggestive of partial neurocognitive independence of SCT from ADHD. Moreover, it points to possibly distinguishable neurobiological neurocognitive underpinnings of the two conditions.

## Introduction

Sluggish Cognitive Tempo (SCT) is a cluster of behaviors that often, but not always, presents in children with Attention Deficit Hyperactivity Disorder (ADHD). This cluster has long been of great interest among ADHD researchers, who initially examined it as a possible defining characteristic of the predominantly inattentive presentation of ADHD, but more recently have considered it as possibly being a separate disorder of attention which is not included in the Diagnostic and Statistical Manual of Mental Disorders-5 (1, 2). Ongoing research has attempted to define and characterize SCT in ADHD and other disorders. One research group proposed up to 150 behavioral items as SCT indicators (3, 4); however, the majority of studies have proposed between four and seven behavioral descriptors that include being slow to arouse, difficulty with maintaining cognitive arousal, being easily confused and having difficulty with rapid processing of information related to a persistent state of dreaminess or sleepiness (3, 5). In efforts to build consensus, a working group recently proposed changing the construct's name to Cognitive Disengagement Syndrome (6, 7) to match their conceptualization of the syndrome, and evolving perspective (2). As the difficulty with naming the construct is a reflection of fluid operational strategies, we chose to examine the construct using the historic lens through which SCT was initially operationalized and distinguished from ADHD inattentive symptoms that are defined in the DSM and refer to attentional control, and so we refer to the syndrome as SCT. Regardless of the name or the criteria used to define it, there is general consensus that SCT is characterized by cognitive and behavioral sluggishness or slowness to alert and arouse attention, deficiencies in maintaining cognitive arousal often seen as high levels of drowsiness, and slow information processing that contributes to being easily confused (4, 7, 8). These domains are captured in the SCT subscale of the Child Behavior Checklist (CBCL) (9), which provides the most cited measure of SCT in secondary analyses of ADHD samples in the extant literature (3).

Existing prospective and retrospective research on SCT indicates a moderate to high correlation (*r* = ∼0.6) with ADHD inattentive symptoms [ADHD-I (3)]. This may be an artifact of the way in which SCT behavioral descriptors are captured, since parents and clinicians who are unfamiliar with ADHD may have difficulty in discriminating behaviors such as daydreaming or seeming spacey from the distractibility and inattention seen in ADHD. However, approximately half of youth with SCT do not have ADHD, and behavioral measures of SCT separate with moderate to high alphas (*α* = .64–.91) from the inattentive symptoms that characterize ADHD when symptom reports are factor analyzed by experienced ADHD researchers and clinicians (3, 10). However, given the potential for subjectivity and overlap in behavioral descriptors, there would be great benefit in characterization of the symptoms through objective measures.

A variety of objective measures have been developed to assess attentional processes (11–13), such as the Continuous Performance Test [CPT (14)] and the Attention Network Test [ANT (15, 16)]. The CPT is one of the most often used and well-validated objective assessment measures of attentional and behavioral control, and while not diagnostic, it has been used to distinguish youth with ADHD from typically developing youth (17). It has long been an important element of clinical testing for academic accommodations (14, 17). Whereas the CPT is designed to test performance limits during attentional challenges, the Attention Network Test (ANT) was developed specifically to map brain systems underlying attentional processes (12, 15, 16, 18, 19). Moreover, the ANT has been used extensively in ADHD research, and the child version (ANT-C) is considered the gold standard neurodevelopmental research tool among youth with ADHD, although it is not used in clinical settings (16, 19–21). Often used in functional brain imaging studies (19, 22), the ANT reliably assesses three distinct attentional networks and their interactions (12, 15, 16, 19, 21, 22)—the Alerting, Orienting, and Executive Control Networks. Given the distinct strengths of each tool in characterizing aspects of attention and attentional performance, their concurrent use had great potential to provide clarity regarding the attentional processes sub-serving the constructs' behavioral descriptors.

The CPT measures response times and accuracy of task performance to calculate *T*-scores of attentional vigilance, distractibility, and impulse control. Although the CPT-II has faced criticism for lacking specificity as a diagnostic tool in distinguishing ADHD from other psychiatric disorders (23, 24), this may reflect the heterogeneous nature of attentional problems in ADHD and other related psychiatric disorders (25). Certainly, there is broad variability in attentional performance among youth with and without ADHD, and the ANT, like the CPT, is not a diagnostic tool. Given that SCT may occur in absence of ADHD and vice versa, associations between ADHD, SCT, and measures of attention are more meaningful with inclusion of youth who had neither ADHD nor SCT, so as to provide insight into the full range of attentional variability and test performance.

To our knowledge, only one study has examined the ANT in measuring SCT (26). This was done in a population sample where youth were not evaluated clinically for an ADHD diagnosis (26). In this study, associations were found for SCT symptoms with correctness of ANT responses and response time, but not with any of the three functional domains assessed by the ANT. A recent comprehensive meta-analysis of nine (9) studies suggested that ADHD was characterized by deficits in the alerting and executive functioning systems, but the inter-reliance of alerting and orienting systems made relative effects difficult to parse (19, 27) and so a method of analysis was developed to account for the inter-reliance (22, 28).

Currently, there is renewed interest in differentiating SCT from ADHD using objective measures of specific components of the attention system. Three literature reviews (2, 5, 10) and a meta-analysis (3) provide a history of the disparate attempts to characterize SCT among samples with ADHD. Solanto et al. (2007) found slower task accomplishment during the Tower of London Task and longer mean reaction times in the CPT (29); and Huang-Pollock et al. (2005) identified problems with early information processing and possible selective attention problems on a perceptual load task (30). Bauermeister et al. (2012), Creque (2021), Jacobson et al. (2018) and Jarrett et al. (2017) found slower processing speed associated with SCT scores (31–34). Skirbekk (2011) found associations between SCT and variability of spatial memory performance among youth with ADHD, but in context of comorbid anxiety disorders (35). Several of these studies [e.g., Solanto (2007)] consider the implications of SCT as potentially being a disorder of executive functioning (specifically a disorder of regulated and integrated attention, rather than a weakness in a particular neural substrate), and the possibility that SCT may differ across age groups as these higher level cognitive systems develop. Given that processing speed is a trans-diagnostic indicator of illness (Rommelse et al., 2020), slower processing is an insufficient measure for capturing and characterizing SCT reliably (36).

The current study was performed with the goal of isolating objective indicators of SCT in a well-characterized sample of youth with ADHD.

Our hypothesis was that greater SCT symptom severity, which is characterized by deficits in the ability to arouse attention and maintain that arousal, would be associated with greater deficits in ANT measures of alerting above and beyond those associated with ADHD alone (37), but not with ANT measures of orienting and executive functioning. CPT results are sensitive but not specific to ADHD. Therefore, we also hypothesized that CPT scores would likely identify the additional attentional burden conferred by SCT. However, we expected that CPT *T*-Scores would correlate with severity of ADHD-I, due the nature of its associated behavioral characteristics.

## Methods

We performed a secondary analysis of pre-treatment (baseline) data derived from two large, NIMH-funded fMRI studies (MH070935 and MH095766) that collected data from 2005 to 2013 and 2013 to 2018, respectively. The goal of these studies was to identify biomarkers of clinical response to methylphenidate or atomoxetine treatment for ADHD. The design of the parent cross-over clinical treatment study associated with MH070935 was described in an analysis of differential medication outcomes assessed via the CPT (38). Study MH095766 was conducted using a parallel group design (39). Both studies used common screening strategies. The primary fMRI finding of differential response used data from the initial R01 dataset has been previously published, and the reanalysis with combined data is in progress (38). All measures, including the version of the ANT developed for use with children (ANT-C) and the CPT were collected using the same standardized procedures. All youth were diagnosed by licensed psychologists and psychiatrists who were expert in ADHD assessment and who both maintained interrater reliabilities and reviewed all cases weekly throughout the studies. Youth were well-characterized with regard to clinical and neurocognitive functioning. All baseline measures, including performance on both the CPT and the ANT, were performed outside the MRI scanner.

### Inclusion/exclusion criteria

Participants were between the ages of 6–17 years, of any gender, sex, race, or ethnicity. Youth in the ADHD group met DSM-IV (and DSM-5) criteria for a primary diagnosis of ADHD Inattentive, Hyperactive, or Combined presentation according to the Kiddie-Schedule of Affective Disorders-PL [KSADS-PL (40)], and had an ADHD-RS score greater than 1.5 SD above the age and sex normed mean for severity in either the ADHD-Inattentive, ADHD-Hyperactive/Impulsive, or ADHD Total domains. Youth could not participate if they met criteria for a current diagnosis, determined by the KSADS-PL, for any other psychiatric disorder except Oppositional Defiant Disorder, dysthymia, or simple phobia. They also could not participate if they had a lifetime history of autism, pervasive developmental disorder, seizure or other neurological disorder, or suicide attempts. Youth with Intellectual Disability (FSIQ < 70) were also excluded. Youth in the healthy control group were matched by age and sex to the youth with ADHD. They could not meet criteria, nor could they experience symptoms at significant subthreshold level for any past or present psychiatric disorders. All youths in this analysis were examined for illicit drug use, and positive findings were exclusionary. Since youth were prescreened for eligibility into the larger treatment study prior to data collection, all youth in this secondary analysis meet those inclusion criteria. Youth with ADHD were physically healthy and willing to participate in medication treatment with either methylphenidate or atomoxetine, and all youth were willing and able to pass safety screening for entering into the fMRI scanning procedures.

Study clinicians who administered diagnostic assessments and clinical interviews were licensed, with extensive experience in ADHD assessment and diagnosis. Research assistants proctored the computer-delivered neuropsychological assessments (ANT and CPT), overseen by trained research and clinical psychologists. All study personnel received standard protocol training in assessment procedures before initiation of the study, and routine inter-rater agreement and protocol fidelity checks were conducted throughout the studies. Interrater reliability across the clinical scales was maintained at >.9.

Between the two studies, a total of *N* = 150 youths ages 6–17 provided assent, following parental consent, to participate. All participants received clinical interviews supported by the KSADS-PL (40); to screen for ADHD and comorbid DSM-IV (and DSM-5) psychiatric disorders. The final participant pool was *N* = 107 youth with ADHD, any type (Inattentive 45.8% or Combined Type 49.5%), and *N* = 30 typically developing controls (*N* = 137), with a mean age of 11.0 (*SD* = 2.8) years. The following measures were used to classify youth for this study.

### Clinical assessments

#### Kiddie-SADS-present and lifetime diagnostic interview [K-SADS-Pl]

The KSADS-PL is a semi-structured interview designed to assess the presence, severity, and course of current and lifetime symptoms of psychiatric disorders among youth (40). The symptom ratings on the KSADS-PL key to DSM-III and DSM-IV diagnostic criteria (1, 41). DSM-5 criteria for diagnosis have not changed substantially from these prior guidelines, and upon review of the records, it was determined that all youth diagnosed in this manner also met criteria for a DSM-5 diagnosis. For youth ages 6–12 years, clinicians rated the KSADS-PL using parent interviews, and incorporated information provided by school and medical records, teacher ratings on behavioral scales, reviews of prior assessment reports, information gleaned from referring care providers, and child self-reports.

#### ADHD rating scale—IV [ADHD-RS]

The ADHD-RS is an 18-item scale that assesses the presence and severity of each of the 18 core symptoms of ADHD (42). Each symptom is scaled from 0 (no symptoms) to 3 (frequent and severe symptoms), and subscale scores from 0 to 27 are derived for both the inattentive and hyperactive/impulsive domains. These subscales are added together to provide a Total ADHD score ranging from 0 to 54, representing overall severity and frequency of symptoms. The ADHD-RS is norm referenced, and provides thresholds determined by age and sex, with 1.5 SD above mean indicating clinically significant elevations in attention problems related to ADHD. In 2016, the ADHD-RS-5 was released with new norms for these domains, and with two additional scales that capture symptom related impairment in each of six functional areas (e.g., home, with homework, in peer relations, behavior at school, academic performance, self-esteem (43). When reviewed against the current norms for the ADHD scales, all participants' scores continued to meet thresholds for inclusion. In this study, The ADHD-RS was administered to parents and adolescents as a clinician rated interview, with prompts for each item. ADHD-I was handled as a dimensional construct, captured as a continuous variable, ranging from the floor of 0 to the ceiling of 27 on the ADHD-RS inattention subscale.

#### Child behavior checklist [CBCL]

The CBCL is a broad-band, age and sex norm-referenced, parent-report inventory designed to assess children's internalizing and externalizing problems, competencies, and DSM-tied symptoms of psychopathology for youth ages 6–17 (44). Since 1988, the CBCL has contained the most widely used norm-referenced scale for characterizing SCT (3). This SCT scale consists of four items that capture the major features of SCT. CBCL item 17 “Lost in his/her thoughts” captures hypo-alertness or variable alertness. CBCL item 80 “Stares blankly” captures the day-time drowsiness and periods of “spaciness” characteristic of SCT. CBCL item 13 “Confused or in a fog” captures the confusion believed to be related to working memory problems in SCT. CBCL item 102 “Underactive, slow moving, lacks energy” captures the low initiative and energy of SCT. SCT subscale *T*-Scores over 70 are considered to be within the clinical range (9), although Barkley (45) uses a less conservative indicator of scoring above the 93rd percentile. *T*-Scores between 65 and 70 are considered to be in the Borderline Range (9). The *T*-Score of the parent reported CBCL (9) provided a dimensional age and sex referenced measure for presence and severity of SCT, ranging from the CBCL floor of *T* = 40 to a ceiling of *T* = 99. As there is currently no diagnostic classification for SCT, the SCT construct was best handled as a continuous variable, using the CBCL thresholds for clinical significance.

#### Wechsler intelligence scales for children—fourth edition [WISC-IV]

The WISC-IV assesses general thinking and reasoning skills of children aged 6–16 years old, and provides a Verbal Comprehension score, Perceptual Reasoning score, Working Memory score, Processing Speed score, and Full Scale IQ score (46). As descriptors of SCT include being slow to process information, or having difficulty processing information as accurately as others, the WISC-IV Processing Speed domain measures have been used to examine SCT behavioral descriptors in context of ADHD, as have working memory problems (47).

#### Attention network test, child version [ANT-C]

The ANT is a modified flanker task that uses Posner's cued attention paradigm (18, 19) to quantify attentional networks, and their interactions (16, 19). Several variations of the ANT-C identify impairments in alerting and executive control in youth with ADHD (16, 19, 37). The recent meta-analysis by Arora et al. (19) identified nine (9) studies that used the ANT-C or a similarly modified ANT with comparable task demands among youth with ADHD through adolescence [e.g., (20, 48)].

This version of the ANT utilizes stimuli that are fish, “swimming” either to the right or left, with flanking fish swimming in the same (congruent) or opposite (incongruent) direction. Participants are directed to “feed the fish” by pressing a right arrow key with their right thumb or index finger, or a left arrow key with a left thumb or index finger. “Feeding” the fish by selecting the correct button elicits a recorded, “Woo Hoo”, to indicate a correct response. An incorrect response elicits a warning tone. A central visual cue precedes the stimulus in cued conditions, but not in uncued conditions. Spatial-cued conditions are preceded by the cue appearing in the location where the stimuli would appear. These result in six possible conditions: no-cue congruent or incongruent, central-cue congruent or incongruent, and spatial-cue congruent or incongruent.

In this study, the ANT was presented in three blocks, each approximately 5 min in duration, with a total task time of approximately 15 min. The assessment was preceded by a teaching trial where youth were able to learn and practice directions for the changing paradigm so as to eliminate confusion about the directions, because we were interested in assessing attention rather than task learning. The method for calculating the three different attention domains is described below in the methods section.

#### Continuous performance test—II [CPT]

Conners' CPT is a computer-delivered task requiring sustained attention, and provides norm referenced *T*-Scores for: missed responses (Omissions), reaction time (Hit Rate), consistency of response time (Hit Rate SE), signal detection (Detectability), response inhibition (Commissions), preservative response style (Perseverance), attentional vigilance (Variability), and response style (Response Bias) (14). It requires sustained attention and adjustment to variable rates of stimulus presentation to test attentional resources among youth with ADHD (14). *T*-Scores greater than 1.5 SD above the mean on each of the outcome measures indicate elevated difficulty with task performance. In this version of the CPT, letters appeared in the center of a screen, and youth were required to press a spacebar for all letters, except an “X” on the screen. The CPT was administered in a standardized manner to derive clinical norms, using the standard six (6) block administration, with three (3) sub-blocks, of 20 trials each. The speed of stimulus presentation varied across presentation blocks, with the entire task lasting approximately 20 min.

### Data analysis

ANT data were examined for validity, skew/kurtosis, and outliers. An additional validity check was performed that involved excluding any ANT scores with a Total Accuracy of <80% of trials (22). No participants' scores met this exclusion criterion. All Response Times were between 200 and 1,500 ms, which is the recommended range for determining whether response times are valid (22). To index the three attentional systems measured in the ANT (Alerting, Orienting, and Executive Control systems), ratios for each of the four cue conditions were calculated (28), using formulas as published in Xiao et al. (22).

All other clinical, cognitive, and behavioral measures were scored according to their standard, published methods, and scores were imported into SPSS 24 for statistical analysis. Demographic and clinical characteristics of the sample were examined, and descriptive statistics were tabulated for reporting. Independent *T*-Tests for Equality of Means were used as pairwise comparisons to determine significant differences in scores between the ADHD and Typically Developing groups on each measure of neuropsychological performance.

Two-tailed Pearson correlation matrices were calculated for all measures. All tests used a significance level of *p* < .05 for exploratory analyses. To control for multiple testing, Bonferroni corrections were calculated by dividing the *p* value by the total number of comparisons in each test's correlation table {*p* corrected = *p*/[*n* correlations*(*n* correlations − 1)/2]}) (49, 50).

## Results

Clinical and Demographic Characteristics are presented in Table 1. The sample demographics, ADHD-RS-IV, and CBCL SCT symptom ratings, and WISC-IV measures of cognitive ability, were examined for the ADHD and Typically Developing groups separately, and combined. Approximately half the sample met criteria for ADHD Combined Presentation (49.5%), and half for ADHD Predominantly Inattentive Presentation (45.8%). The overall sample was 22.4% (*n* = 24) White, 27.1% (*n* = 29) African American, 0.9% (*n* = 1) Asian, 5.1% (*n* = 7) Multi Racial Non-Hispanic, 43% (*n* = 46) Multi Racial Hispanic. Forty three participants with ADHD (40.1%) met criteria for Oppositional Defiant Disorder (ODD), which is highly comorbid with ADHD (1). Demographic variables, including ADHD phenotype (e.g., hyperactivity/impulsivity), common comorbidity such as ODD/CD, sex, age, race, ethnicity, and SES were not different across groups or measures of interest. There were no differences in gender distribution across groups [*χ*^2^(1,137) = 1.268, *p* = .260], and no gender differences in SCT scores (Mann–Whitney *U* = 218.5, *p* = .830).

**Table 1:** Sample demographics and behavioral measures.

	Age *M* (SD)	Male *N* %	Hispanic *N* %	ODD *N* %	ADHD-RS total *N* (SD)	ADHD-RS-I (SD)	ADHD-RS-H/I (SD)	CBCL SCT-T (SD)	WISC-IV PSI (SD)	WISC-IV WMI (SD)	WISC-IV VCI (SD)	WISC-IV PRI (SD)	WISC-IV FSIQ (SD)
ADHD *N* = 107	11.2 (2.9)	79 73.8%	46 42.9%	43 40.2%	37.6 (10.4)	22.2 (4.6)	15.4 (7.9)	65.3 (7.3)	83.7 (22.6)	93.7 (18.2)	101.3 (15.8)	98.8 (15.3)	98.7 (14.7)
Control *N* = 30	12.4 (3.0)	19 63.3%	8 26.6%	0	1.3 (2.2)	0.9 (1.7)	0.4 (0.8)	50.9 (2.1)	81.2 (18.2)	103.9 (14.0)	107.8 (13.3)	103.4 (12.3)	113.3 (17.1)
Total *N* = 137	11 (2.8)	98 71.5%	54 39.4%	43 31.4	29.7 (17.6)	17.6 (9.8)	12.1 (9.4)	59.8 (9.2)	83.2 (21.8)	95.7 (17.8)	102.5 (15.5)	99.8 (14.8)	102 (16.4)

ODD, oppositional defiant disorder diagnosis; ADHD-RS Total, ADHD-RS-IV total score; ADHD-RS-I, ADHD-RS-IV inattentive subscale score; ADHD-RS-H/I, ADHD-RS-IV hyperactivity/impulsivity subscale score; CBCL SCT-T, child behavior checklist sluggish cognitive tempo scale *T*-score; WISC-IV PSI, Wechsler intelligence scale for children IV processing speed index score; WISC-IV WMI, Wechsler intelligence scale for children IV working memory index score; WISC-IV VCI, Wechsler intelligence scale for children IV verbal comprehension index score; WISC-IV PRI, Wechsler intelligence scale for children IV perceptual reasoning index score; WISC-IV FSIQ, Wechsler intelligence scale for children IV full scale IQ score.

Task Performance for the youth with ADHD compared to typically developing youth is presented in Table 2. Independent samples *T*-Tests for Equality of Means for performance on the CPT-II and ANT tasks are presented, as are pairwise comparisons to determine significant differences in scores between the ADHD and Typically Developing groups. Significant mean differences were found between the ADHD and Typically Developing groups on the CPT-II for errors made (Commission Errors *t* = 3.217, *p* = .002, Mean Difference = 6.69), missed responses (Omission Errors *t* = 2.950, *p* = .004, Mean Difference = 9.73), perseverative response style (Perseverance *t* = 3.249, *p* = .001, Mean Difference = 10.91, variability in speed of response (Hit Rate SE *t* = 3.256, *p* = .001, Mean Difference = 8.11), and variability in performance over time (Variability *t* = 4.177, *p* < .001, Mean Difference = 9.81). Significant mean differences were found between the ADHD and Typically Developing groups on the ANT measure of Executive Control (EC *t* = 2.984, *p* = .004, Mean Difference = .054).

**Table 2:** Neurocognitive task performance in youth with ADHD and typically developing youth and youth with high and low SCT scores.

	ADHD		TD					
	*M*	SD	*M*	SD	*t*	*df*	*p*	*M diff*
CPT commissions *T*	52.97	8.99	46.282	11.333	3.217	121	.002*	6.692
CPT detection *T*	53.369	9.344	49.563	12.604	1.724	121	.087	3.806
CPT hit rate *T*	47.877	12.310	47.783	11.749	.035	121	.972	.094
CPT hit rate SE *T*	55.668	11.641	47.563	10.603	3.256	121	.001*	8.105
CPT omissions *T*	56.409	16.758	46.673	6.518	2.950	121	.004*	9.737
CPT perseverance *T*	58.872	17.013	47.957	7.001	3.249	121	.001*	10.915
CPT response bias *T*	52.474	10.163	52.220	14.866	.103	121	.918	.254
CPT variability *T*	55.970	10.793	46.161	10.735	4.177	121	.000*	9.810
ANT alerting RT	.056	.080	.047	.054	.560	99	.577	.009
ANT orienting RT	.054	.087	.045	.063	.506	99	.614	.009
ANT ex. control RT	.106	.085	.052	.068	2.984	99	.004*	.054
	SCT >70		SCT <70					
	*M*	SD	*M*	SD	*t*	*df*	*p*	*M diff*
CPT commissions *T*	53.903	7.919	49.474	10.653	−1.757	94	.042	−4.43
CPT detection *T*	53.635	7.619	51.379	11.096	−0.876	94	.192	−2.26
CPT hit rate *T*	45.204	12.304	46.443	11.171	0.411	94	.341	1.24
CPT hit rate SE *T*	52.799	11.043	49.165	11.095	−1.265	94	.105	−3.63
CPT omissions *T*	52.336	13.075	48.667	8.845	−1.293	94	.100	−3.67
CPT perseverance *T*	55.571	11.628	52.064	14.242	−1.015	94	.157	−3.51
CPT response bias *T*	48.828	7.284	51.114	12.740	0.801	94	.213	2.29
CPT variability *T*	52.748	11.138	48.025.05	11.521	−1.600	94	.057	−4.72
ANT alerting RT	.094	.080	0	.062	−2.445	73	.009*	−0.04
ANT orienting RT	.045	.106	.045	.065	0.000	73	.500	0.00
ANT ex. control RT	.106	.074	.080	.078	−1.299	73	.099	−0.03

CPT, continuous performance test; ANT, attention network test; *T*, *T* score.

* Significant at <.005.

Correlations between scores of youth with ADHD-I and ADHD + SCT on tests of cognitive ability, and on neuropsychological task performance are presented in Table 3. Spearman correlations between scores of youth with ADHD-Inattention symptoms and those with ADHD + SCT are presented along with those of cognitive ability as assessed by the WISC-IV. ADHD-I symptoms and SCT *T*-scores were highly inter-correlated (*r*^2^ = .806, *p* < .001); however, only ADHD-I and not SCT was associated with WISC-IV Full Scale IQ (*r*^2^ = −.362, *p* < .001) after Bonferroni correction. An association between the WISC-IV Working Memory Index and ADHD-I did not retain significance upon Bonferroni correction (*r*^2^ = −.238, *p* = .014). SCT was not associated with the WISC-IV Full Scale IQ or any WISC-IV domain scores, either before or after Bonferroni correction.

**Table 3:** Associations between ADHD-I, SCT, and WISC-IV.

		ADHD-I	SCT	FSIQ	VCI	PRI	WMI
SCT	*R*	.806**					
	(*p*)	(<.000)					
FSIQ	*R*	−.362**	−.271				
	(*p*)	(<.000)	(.022)				
VCI	*R*	−0.160	−0.116	.723**			
	(*p*)	(.090)	(.382)	(<.000)			
PRI	*R*	−0.165	−0.078	.679**	.544**		
	(*p*)	(.113)	(.642)	(<.000)	(<.000)		
WMI	*R*	−.238**	−0.187	.431**	.364**	.472**	
	(*p*)	(.014)	(.159)	(<.000)	(<.000)	(<.000)	
PSI	*R*	.000	0.238	.218	.210	.408**	.407**
	(*p*)	(.996)	(.072)	(.021)	(.027)	(<.000)	(<.000)

SCT, sluggish cognitive tempo; FSIQ, full scale IQ; VCI, verbal comprehension index; PRI, perceptual reasoning index; WMI, working memory index; PSI, processing speed index.

** Retains significance after Bonferroni correction.

Table 4 presents associations between ADHD-I, SCT, and CPT-II performance. CPT scores reflecting errors made in task performance were significantly correlated with ADHD-I, with higher ADHD ratings correlating with more errors of both Omission and Commission (Commission Errors *r*^2^ = .282, *p* = .002; Omission Errors *r*^2^ = .254, *p* = .005). These associations remained significant after correction for multiple comparisons. Neither Commission nor Omission errors correlated with SCT behavioral symptoms. Variability in speed of response (Hit Rate SE *r*^2^ = .272, *p* = .002), and variability in performance over time (Variability *r*^2^ = .328, *p* < .001) also retained significance in correlations with ADHD-I, after Bonferroni corrections.

**Table 4:** Associations between ADHD-I, SCT, and CPT-II.

		ADHD-I	SCT	Commission	Detect	HR	HR SE	Omission	Persev	Resp bias
Commissions	*R*	.282**	0.101							
	(*p*)	(.002)								
Detectability	*R*	.177	0.029	.801**						
	(*p*)	(.055)		(<.000)						
Hit rate	*R*	.004	−0.087	−.370**	−0.154					
	(*p*)			(.000)						
Hit rate SE	*R*	.272**	0.051	0.165	0.151	.645**				
	(*p*)	(.003)				(<.000)				
Omissions	*R*	.254**	0.073	0.022	0.173	.597**	.745**			
	(*p*)	(.005)			(.056)	(<.000)	(<.000)			
Perseverance	*R*	.205	0.046	.348**	0.151	0.159	.552**	.297**		
	(*p*)	(.026)		(<.000)			(<.000)	(.001)		
Response bias	*R*	−.018	−0.074	−0.067	0.110	.418**	.447**	.425**	.191	
	(*p*)					(<.000)	(<.000)	(<.000)	(.035)	
Variability	*R*	.328**	0.087	.254	.196	.494**	.917**	.687**	.510**	.391**
	(*p*)	(<.000)		(.005)	(.030)	(<.000)	(<.000)	(<.000)	(<.000)	(<.000)

** Retains significance after Bonferroni correction.

Table 5 presents the associations between ADHD-I, SCT, and performance on the ANT. The ANT Alerting index was significantly correlated with SCT symptoms, with better alerting associated with less SCT symptom severity (*r*^2^ = −.291, *p* = .005). No other ANT attention indices were associated with either SCT or ADHD-I. ANT attention scores were positively correlated with one another, and with process factors of the CPT in that better attentional performance on the ANT was associated with better performance on the CPT (fewer errors, less variability of attention, and better response times; see Table 6).

**Table 5:** Associations between ADHD-I, SCT, and ANT.

		SCT	ADHD I	ANT alerting	ANT orienting
Alerting	*r*	−.291	−.063		
	*p*	.005	.540		
Orienting	*r*	.139	.057	−.482**	
	*p*	.192	.580	0.000	
Executive control	*r*	−.001	.162	0.149	−.253*
	*p*	.993	.116	0.138	0.011

** Retains significance after Bonferroni correction.

**Table 6:** Associations between ANT and CPT.

		Commission	Detectability	HR	HR SE	Omissions	Perseverance	Bias	Variability
Alerting	*R* ^2^	0.057	0.096	−.278**	-.239*	-.212*	−0.094	−0.073	−0.189
	*p*	0.577	0.346	0.005	0.017	0.035	0.355	0.473	0.061
Orienting	*R* ^2^	−0.038	−0.119	.300**	.288**	.230*	.366**	0.090	.229*
	*p*	0.707	0.242	0.003	0.004	0.022	0.000	0.374	0.023
Exec Ctrl	*R* ^2^	0.063	−0.017	−0.167	0.036	0.051	0.059	0.065	0.082
	*p*	0.533	0.864	0.099	0.721	0.615	0.561	0.520	0.419

* = *p* <.05, **Retains significance after Bonferroni correction.

## Discussion and conclusions

There has been a great deal of interest in finding behavioral descriptors to characterize the construct of SCT within the ADHD population, but there has been little work done to characterize SCT using objective measures. We sought to extend our knowledge about the neurocognitive correlates of ADHD and SCT using a well characterized clinical sample and typically developing youth. We did so with a well-validated research tool (the ANT) and a well-validated neuropsychological task that is frequently used in clinical evaluation (the CPT). To our knowledge, this is the first study to use both the ANT and the CPT-II to examine potential similarities and differences in neurocognitive function between ADHD and SCT in a clinically diagnosed sample, including typically developing comparators. Although the alerting measure was a relatively modest contributor to SCT reports, the association was strong enough to hold up under very conservative testing methods. Similarly, the association of attentional performance and control with ADHD was strong enough to remain after conservative procedures. Together, these findings form a double dissociation that indicates objective neurocognitive measures of attention may be useful in identifying and describing differences between the ADHD and SCT constructs that may be difficult to parse using behavior ratings alone (Barkley, 2016; Becker, 2016).

ADHD-I ratings were expectedly correlated with SCT ratings, since the two conditions are known to be related. The typically developing control group in this study would not be expected to have high levels of ADHD or SCT symptoms. Our small but noteworthy double dissociation between ADHD + SCT and ADHD-SCT on neurocognitive measures of attention remained stable even after conservative methods were used to account for these correlations.

Objective neurocognitive performance diverged on two separate attention tasks for individuals, based on their higher symptom ratings of ADHD-I as compared to those with higher SCT ratings. Youth with higher SCT scores had more difficulty with attentional alerting than did youth with lower SCT scores, suggesting that SCT may represent deficits in an early stage of attention during which information is passively filtered through the sensory system to determine salience and subsequent arousal of consciousness toward salient information. Youth with lower SCT scores, performed well on attentional alerting, but had difficulty with maintaining consistent attentional control over time, during a simple and boring task, as is consistent with ADHD. These differences in performance suggest there may be different neuropsychological underpinnings for the two behavioral constructs, and that they are at least partially distinct. These findings also provide objective support for parent-report scales indexing the behavioral descriptors for SCT, from which our hypothesis originated: being slow to arouse and being cognitively sluggish. Additional study should examine whether these findings remain consistent in other samples and across other granular tests of neurocognitive abilities. In a study in press in this issue, that used a standardized computer delivered neurocognitive test battery to examine adults with ADHD + SCT and ADHD-SCT, SCT was associated with subtle neurocognitive deficits on tasks requiring visual spatial skills similar to those of the ANT. However, in that study, adults with SCT performed worse with increasing cognitive loads. Since there is some indication that executive functioning may play a larger role in SCT presentation among older teens and adults, it is important to examine SCT's development across the lifespan.

In this sample, behavioral reports of SCT, including being slow to arouse or cognitively sluggish, were only captured on objective measures of arousal, but not visual-motor measures of processing speed. This suggests that youth with SCT were able to process information as quickly as other youth with ADHD once they were alert and engaged in the task. SCT was not associated with auditory working memory in this cohort, although weak working memory is often an SCT descriptor. SCT was also not captured by performance on CPT measures of speed (response time) or ability to recognize targets (detectability) despite SCT descriptors including being slow and being easily confused. These measures indexed only ADHD. While the CPT does not provide an index of attentional alerting or arousal, the latter was somewhat unexpected, since CPT scores are non-specific indicators of attention problems among youth with broad psychopathology and SCT is generally associated in the literature with high rates of co-occurring disorders (23, 24). In this sample, mood and anxiety disorders requiring treatment, autism spectrum disorders, seizure disorders, uncontrolled sleep disorders, and other health problems that affect attentional control to conflate with ADHD on CPT tests were exclusionary (51), and in absence of these disorders SCT was not conflated. The enduring dissociation of SCT and ADHD on arousal mechanisms was also somewhat unexpected, since while we hypothesized a stronger association for SCT with arousal, ADHD also tends to associate with arousal deficits (19, 37).

Our findings are somewhat divergent from others. However, prior reports of an association between increased response times on CPT and the presence of SCT behavioral indicators may be an artifact of sampling. In one study, all participants were diagnosed with ADHD Inattentive presentation while our sample examined SCT among youth with both ADHD Inattentive and Combined presentations (29). Features of impulsivity and hyperactivity may mask associations between CPT performance and SCT. In another study, SCT severity was associated with increased response time, but across a battery of measures with increasing complexity and cognitive load (32). This suggests that SCT may manifest not only on specific challenge of attentional arousal, but also on tests with increasing cognitive load. In this way, SCT may manifest as an executive dysfunction apart from response inhibition and executive control of attention—which the CPT specifically tests.

The findings of this study are in partial agreement with the literature examining similarities and differences between ADHD-I and SCT using parent ratings, which generally finds dissociation of the two constructs (Becker et al., 2019). Almost two-thirds of our youth with clinically diagnosed ADHD also had clinically elevated SCT symptoms. We found a stronger association between ADHD-I and SCT which may be consistent with clinical construct overlap (Becker, 2016; Becker et al., 2019). Interdependence of attention networks and shared neurobiological underpinnings of attentional processes (Wand et al., 2014) may explain observed clinical overlap between ADHD-I and SCT.

There are several limitations to the study. SCT was defined by parent-reported SCT behavioral descriptors using the CBCL SCT subscale. While data from teachers regarding ADHD were collected and informed parent reports of ADHD, cross-domain manifestations of SCT behaviors were not captured from teacher reports on the teacher version of the CBCL, nor were other, more recently developed SCT scales used. While the CBCL 4-item proxy that was used is considered an adequate and valid norm-referenced measure of SCT behaviors (Willcut et al., 2014), newer scales exist to more fully explore this experimental construct. In addition, although the sample size was large enough to provide reasonable assurance of statistical power, a larger sample recruited to specifically examine behavioral features of SCT would have allowed for a more focused exploration of moderators of SCT. Inclusion of youth selected for SCT in the absence of ADHD would have offered additional opportunity to assess the SCT construct independently from ADHD, and possibly offer a better opportunity to discriminate processes distinctly related to the two conditions. Finally, this was a secondary analysis.

Despite these limitations, the finding of a double dissociation in this study—with ADHD-I symptom severity related to measures of cognitive and behavioral control measures on the CPT, and SCT symptom severity related to the alerting/arousal measure on the ANT—provides the first objective evidence of partial neurocognitive independence of SCT from ADHD. Moreover, it points to possibly distinguishable neurocognitive underpinnings of the two conditions. Among typically developing individuals, CPT performance invokes frontal, temporal, occipital, and anterior cingulate cortices (ACC), the insula, and the cerebellum (52). The ACC and the cerebellum each play central roles in the selective and focused attention on CPT performance (12, 51, 52), and the ACC and medial frontal cortex are involved in the cognitive monitoring and evaluation required to detect errors of performance (51, 52). The invocation of the frontal cortex (specifically the lateral prefrontal cortex) and the ACC underlie the inhibitory control and conflict resolution processes that are central in the ANT executive control domain (15, 22, 53). Thus, the CPT may be a more sensitive or specific measure of executive control among our sample of youth with ADHD whereas the ANT alerting system, which was associated with SCT symptoms in our sample, may be a more specific measure of attentional processes underlying tonic arousal in preparation for cue detection. Specifically, the alerting domain invokes right frontal and parietal brain regions associated with noradrenergic function in the locus coeruleus (15, 22), which is a target mechanism for several of the noradrenergic drugs used to treat ADHD.

These results provide a foundation for future studies to objectively characterize the relationship of SCT and inattentive symptoms of ADHD, and more conclusively examine the extent to which SCT and ADHD are truly distinct constructs. Future research is needed to examine whether SCT impacts the course or outcome of ADHD treatment, and whether different ADHD treatments have preferential effects in one or the other condition. As we more fully understand the mechanisms underlying SCT, we can more appropriately develop treatments for the impairments with which it is associated.

## Data Availability

The raw data supporting the conclusions of this article will be made available by the authors, without undue reservation.
